# Bereaved parents’ perceptions of memory making: a qualitative meta-synthesis

**DOI:** 10.1186/s12904-024-01339-0

**Published:** 2024-01-25

**Authors:** Dan-dan Xu, Guang-xiong Zhang, Xin-bo Ding, Jing Ma, Ya-xi Suo, Yang-yao Peng, Ji-li Zeng, Miao Liu, Rui-tong Hou, Jin Li, Fen Hu

**Affiliations:** 1 Department of Critical Care Medicine, Zhongnan Hospital of Wuhan University, Clinical Research Center of Hubei Critical Care Medicine, PO Box 430071, No. 169 Donghu Road, Wuhan, Hubei Province China; 2 Department of Anesthesiology, Hubei Province Corps Hospital of The Chinese Armed Police Force (CAPF), Wuhan, China; 3 Department of Neurosurgical Intensive Care Unit, Zhongnan Hospital of Wuhan University, Wuhan, China; 4 Department of Otorhinolaryngology, General Hospital of Central Theater Command, Wuhan, China; 5 Department of Pulmonary Oncology, Zhongnan Hospital of Wuhan University, Wuhan, China

**Keywords:** Bereaved parents, Memory making, Qualitative study

## Abstract

**Objective:**

This study aims to investigate the experiences of parents who have experienced bereavement in their efforts to preserve memories of their deceased child.

**Methods:**

Employing a qualitative meta-synthesis approach, this study systematically sought relevant qualitative literature by conducting searches across various electronic databases, including PubMed, Embase, CINAHL, PsycINFO, Web of Science, Cochrane Library, and Wiley, up until July 2023.

**Results:**

Nine studies are eligible for inclusion and included in the meta-synthesis. Three overarching categories are identified: (1) Affirming the Significance of Memory Making. (2) Best Practices in Memory Making. (3) Barriers to Effective Memory Making.

**Conclusion:**

Bereaved parents highly value the act of creating lasting memories, emphasizing its profound significance. While forming these memories, it is imperative to offer family-centered care and honor diverse preferences and needs. It is essential to offer effective support to parents, offering them a range of choices. Furthermore, a more comprehensive examination of memory-making practices is required to better understand their influence on parents’ recollections of their deceased child.

**Supplementary Information:**

The online version contains supplementary material available at 10.1186/s12904-024-01339-0.

## Introduction

The loss of a child profoundly impacts grieving parents and may result in significant adverse outcomes [1]. Globally, in 2021, infants faced the highest mortality risk within their first month of life, with an average rate of 18 deaths per 1,000 live births [2]. Bereaved parents often experience substantial distress, manifesting as physical and emotional symptoms, including fatigue, insomnia, pain, sadness, helplessness, and guilt, among others [3, 4]. Furthermore, parental suicide and marital breakdown may result from the loss of a child [5]. Women demonstrate a higher propensity for experiencing suicidal ideation subsequent to the loss of a child in comparison to men [6]. Grief can have a profound effect on an individual’s immune system, leading to increased susceptibility to heart attacks and overall mortality when parents experience the loss of a child [7]. Moreover, they face a higher risk of depression, anxiety, and post-traumatic stress disorder in comparison to parents who have not undergone such an ordeal [7, 8]. Within intensive care units, children often experience isolation, which significantly reduces parental interaction. This lack of engagement limits recollections and opportunities for parental involvement, thereby increasing the risk of persistent complex bereavement disorder or complex grief among parents [9]. The self-awareness and isolation experienced by parents can exacerbate their feelings of sadness [10]. Hence, there is an urgent need to implement effective coping interventions for parents and caregivers coping with the loss of a child.

Identifying parents’ grief needs at the time of a child’s death is a fundamental step in providing appropriate care. An expanding body of research is dedicated to exploring grief programs within neonatal intensive care units (NICU) [11], the requirements of grieving parents [12], and nurses’ comprehension and viewpoints regarding bereavement services [13]. Numerous bereavement practices and parent- centered interventions have emerged, encompassing activities like legacy formation, support groups, family-centered companionship and post-loss support, parental participation in pre-mortem care, intergenerational bereavement programs, and the utilization of technological and spiritual resources [14]. These approaches contribute to parents feeling supported, diminishing their sense of isolation, and enhancing their coping abilities. Despite guidelines recommending bereavement support for families, the absence of a standardized program presents challenges in implementation [15]. Kochen’s review of parent-centered bereavement interventions indicates that the absence of follow-up contact from medical staff may lead parents to perceive abandonment, potentially exacerbating their feelings of loss [16]. Bereavement support should concentrate on the enduring nature of grief and should commence before the child’s death to assist parents in navigating the transitional phase following their loss.

Memory-making practices are gaining recognition as a means to address the bereavement needs of parents and their families. Memory making primarily entails parents’ time spent with their stillborn child, encompassing activities such as holding, washing, and dressing the deceased infant [17]. Numerous children’s hospitals provide legacy building projects for grieving parents [18, 19], typically involving painted handprints, photographs, and plaster hand casts. Caregivers and child life specialists commonly oversee these projects [20, 21]. Memory making can offer tangible mementos for family members, facilitate emotional expression, alleviate grief, and foster a connection between parents and their departed children. Research has highlighted the significance of late-infant physical interactions. Families can create and collect tangible mementos of their children to aid in the grieving process [22]. Barrett’s research suggests that families highly value the ability to maintain a connection with their deceased children and stay emotionally close to them [23]. The absence of such contact can be a source of parental grief; however, not all parents avail themselves of the chance to forge meaningful memories. Various modalities have been identified as avenues for memory creation interventions. Nevertheless, there is limited knowledge regarding parents’ sentiments and perspectives on engaging in memory-making practices. Especially in different cultural contexts, parents have different views on memory making. It is necessary to elucidate parental needs and formulate efficacious bereavement interventions.

The objective of this study is to gain insight into parents’ experiences with memory making, ascertain their perspectives on the practice, and investigate the determinants influencing the implementation of memory making. The research questions of this study are as follows:

1. What are parents’ perspectives regarding memory making?
2. What factors exert influence on memory making?

## Methods

### Search strategy

To fulfill this objective, a comprehensive literature search was conducted to identify all qualitative studies focused on exploring bereaved parents’ perceptions and emotions concerning memory making. The search encompassed multiple databases, including PubMed, Embase, CINAHL, PsycINFO, Web of Science, Cochrane Library, and Wiley, in July 2023. The search terms utilized were: “hospice care”, “terminal care”, “parents”, “father”, “mother”, “intervention”, and “strategy”.

### Inclusion and exclusion criteria

Inclusion criteria: (1) The study design incorporated a qualitative approach, encompassing the qualitative segment of the mixed-method study. (2) With the primary participants being bereaved parents. (3) The incorporation of one or more memory-making interventions. Exclusion criteria: (1) Unavailability of the full-text literature. (2) Literature not published in English. (3) Duplicate publications or incomplete information.

### Data extraction

The search results were imported into NoteExpress. Researchers familiarized themselves with the study content through multiple separate readings. Subsequently, eligible data were extracted into a Microsoft Excel spreadsheet, encompassing information such as the study’s purpose, participant characteristics, data collection methods, and research approaches. Any discrepancies were resolved through discussion and evaluation, with the involvement of a third examiner if required.

### Quality appraisal

Two researchers utilized the CASP qualitative research checklist to evaluate the quality of the included articles [24]. Any discrepancies were resolved through discussion and, if necessary, referred to a third reviewer. In qualitative synthesis, the relevance of topics takes precedence over the quality of individual studies, making it inappropriate to exclude studies solely due to quality concerns. Despite minor variations in the quality of the included studies, none of them exhibited such poor reliability as to preclude their use in the synthesis.

### Data synthesis

We employed thematic synthesis [25], consisting of the following steps: (1) Line-by-line coding of relevant textual data. (2) Organization of codes into descriptive themes. (3) Analysis of theme development. We analyzed the original data from each individual study included in this research. We thoroughly examined topic or category names, explanations, and interpretations provided by the original authors, as well as statements made by study participants concerning each topic. Concurrently, we conducted an analysis of the original data from each included study. Building upon these summarized results, we conducted a comprehensive reanalysis by comparing similarities and differences among the findings, as well as assessing the diversity across the studies.

## Results

### Search result

**Fig. 1:**
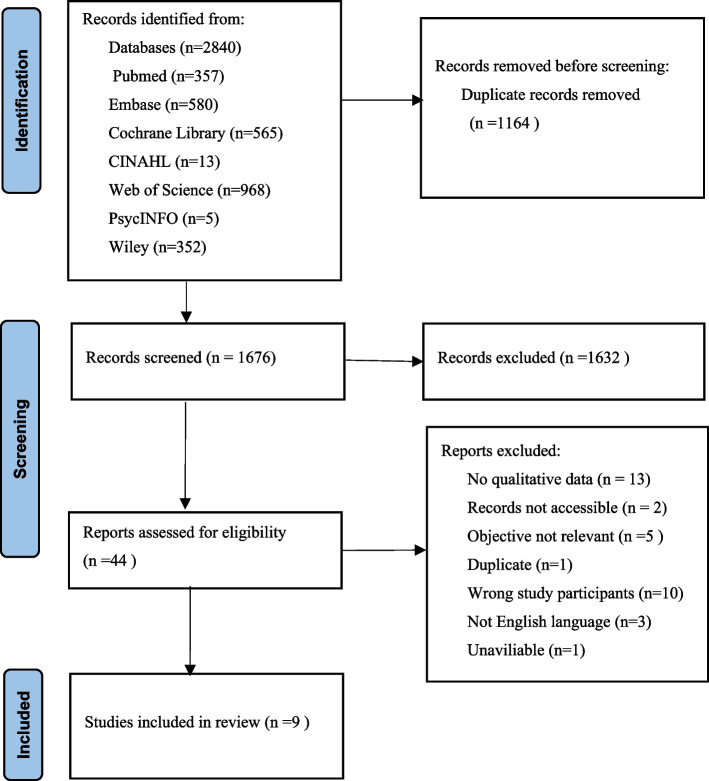
Flow diagram of studies included and excluded at each stage of review

Figure 1 shows the screening process. The predominant reason for excluding studies during the title and abstract screening phase was their non-alignment with the defined criteria regarding study populations and designs. During the full-text screening, the primary basis for exclusion was the absence of parents who had undergone memory making experiences.

### Characteristics of the included studies

The nine included studies employed a range of methodological approaches, displaying heterogeneity. These approaches encompassed grounded theory [22, 26] (*n* = 2), phenomenological description [27–29] (*n* = 3), and content analysis [19, 30–32] (*n* = 4). The majority of these studies employed thematic analysis to extract themes. However, in our study, we selectively extracted expressions solely from bereaved parents, omitting healthcare providers and photographers from the analysis [19, 26, 29]. Furthermore, one study gathered experiences and perspectives of bereaved parents through an online questionnaire and subsequently analyzed the data using qualitative research methods, warranting its inclusion [32]. Detailed characteristics of the included studies are provided in Table 1. It is noteworthy that the majority of interviewees in these studies are mothers.

**Table 1:** Summary of included studies

**Author (first), year**	**Country**	**Aim**	**Setting**	**Participants**	**Children’s diseases and developmental stages**	**Methods**	**Findings (Themes)**
Clarke [27] (2022)	Ireland	To explore the lived experience a memory making process have on parents of children who are at or near end-of-life.	In a hospice	6 mothers	Infants and children	semi-structured interviews/Interpretative phenomenological analysis	1. Making the memories. 2. The impact of memory making. 3. The end-of-life care journey.
Love [30] (2022)	USA	To characterize bereaved parents’ perspectives on the value of legacy activities; to describe parent recommendations for optimizing provision of legacy activities by child life specialists and music therapists.	Phone interview	17 mothers, 2 fathers	Children dying of cancer	semi-structured interviews/content analysis	1. The value of legacy items and interventions. 2. The practical roles, uses, and functions of legacy items. 3. Best practices for offering legacy interventions.
Goldberg [28] (2022)	USA	To understand the legacy experiences and perceptions of parents who have experienced perinatal or early infant (less than 3 months of age) loss.	Individual in-person, phone interview, and Zoom videoconferencing	7 mother, 3 fathers	Perinatal and early infant (less than 3 months of age)	unstructured interview/ Phenomenological qualitative traditions	1. Legacies are composed of memories and experiences that have a lasting effect on others. 2. Healthcare experiences both generate and participate in infants’ legacies. 3. Parents’ legacy perceptions are shaped by cultural conceptions, spiritual beliefs, and grief experiences.
Thornton [22] (2021)	Australia	To explore the significance of memory-making for bereaved parents and the impact of memory-making on parents’ experience of neonatal end-of-life care.	Telephone, Skype and face-to-face interviews	13 mothers, 5 fathers	Infants	Ground theory	1. Creating Evidence 2. Needing Guidance 3. Being a parent
Schaefer [19] (2020)	USA	To explore the legacy-making and grief experiences of bereaved parents who participated in legacy artwork with their child before his or her death from cancer	Individual in-person and phone interview	6 mothers, 6 fathers	Children dying of cancer	semi-structured interviews/content analysis	1. Legacy artwork allows for family bonding and opens communication regarding the child’s impending death. 2. Provides opportunities for parents to engage in life review and meaning-making, is often displayed in the parents’ home after the child’s death. 3. Parents take comfort in using these projects to continue their bond with their deceased child. 4. Can ameliorate parents’ grief after their child’s death. 5. May reduce healthcare providers’ compassion fatigue and provide them an outlet for coping with their patients’ deaths.
Ramirez [26] (2019)	USA	To understand the role of professional bereavement photography in assisting the grieving process of parents who have lost a fetus or infant.	Phone interview	4 mothers, 2 fathers	Stillbirths and infants	Grounded theory	1. Validation of the experience 2. Permission to share. 3. Creation of a permanent and tangible legacy. 4. Creation of positive memories. 5. Moving forward after the loss.
Akard [31] (2018)	USA	To explore bereaved parents’ perceptions of a digital storytelling legacy-making intervention for parents after the death of an infant.	In a private living room style conference room.	3 mothers, 3 fathers	Infants	Focus group/content analysis	1. Parents’ willingness to participate in a legacy intervention. 2. Parents’ suggestions for a feasible intervention. 3. Parents’ suggestions for an acceptable intervention. 4. Parents’ perceived benefits of legacy-making.
Blood [32] (2014)	USA	To explore the patents experience to ostmortem memento photography.	online survey	101 mothers, 1 fathers	Perinatal death	content analysis	1. Obstacles to postmortem photography. 2. Supporting parents’ needs. 3. Creating quality mementos. 4. Creating quality mementos. 5. Parents who were not asked if they wanted photos. 6. Parents who do not have pictures. 7. Broad appreciation expressed by parents.
Martel [29] (2014)	Canada	To explore parents experience End-of-Life (EOL) photography around the death of their newborn in the neonatal intensive care unit (NICU) and in their lives beyond the hospital	At the hospital	6 mothers, 4 fathers	Newborn	semi-structured interviews/Phenomenological Study	Parenting and knowing

### Quality appraisal

The majority of the studies exhibited high quality and fulfilled several of the CASP quality criteria, as summarized in Table 2. Nevertheless, a noteworthy limitation across most of the included studies was the absence of adequate documentation concerning the relationship between researchers and interviewees. This limitation posed a challenge to the overall quality of the research.

**Table 2:** Methodological assessment

	**Q1**	**Q2**	**Q3**	**Q4**	**Q5**	**Q6**	**Q7**	**Q8**	**Q9**	**Q10**
Clarke [27] (2022)	Y	Y	Y	Y	Y	U	Y	Y	Y	Y
Love [30] (2022)	Y	Y	Y	Y	Y	Y	Y	Y	Y	Y
Goldberg [28] (2022)	Y	Y	Y	Y	Y	U	Y	Y	Y	Y
Thornton [22] (2021)	Y	Y	Y	Y	Y	U	Y	Y	Y	Y
Schaefer [19] (2020)	Y	Y	Y	Y	Y	U	Y	Y	Y	Y
Ramirez [26] (2019)	Y	Y	Y	Y	Y	Y	Y	Y	Y	Y
Akard [31] (2018)	Y	Y	Y	Y	Y	Y	Y	Y	Y	Y
Blood [32] (2014)	Y	Y	Y	Y	Y	U	Y	Y	Y	Y
Martel [29] (2014)	Y	Y	Y	Y	Y	U	Y	Y	Y	Y

The CASP tool assesses the quality and usefulness of research studies with a ten-question survey. The questions are:

Q1=Was there a clear statement of aims?

Q2=Is the methodology appropriate?

Q3=Was the research design appropriate to address the aims?

Q4=Was the recruitment strategy appropriate to the aims of the research?

Q5=Were the data collected in a way that addressed the research issue?

Q6=Has the relationship between the researcher and participants been considered?

Q7=Have ethical issues been taken into consideration?

Q8=Was the data analysis sufficiently rigorous?

Q9=Is there a clear statement of findings?

Q10=How valuable is the research?

*U* Unclear, *Y* Yes

### Meta‐synthesis of qualitative date

We synthesized and identified three overarching themes along with eleven corresponding subthemes. These three central themes encompass: (1) Affirming the Significance of Memory Making. (2) Best Practices in Memory Making. (3) Barriers to Effective Memory Making.

### Affirm the value of memory making

#### Contact

Parents underscored the significance of memory making, highlighting that crafting legacy artwork facilitated quality time spent together. They expressed contentment with possessing photographs and handmade representations of their children, facilitating an enduring connection as these tangible objects provided a constant reminder. Simultaneously, these mementos offered a platform for sharing narratives and cherished memories with both relatives and friends.

##### The legacy of memory creation helps parents establish an ongoing tangible connection with their children

*“They’re just more tangible, they’re more real [pause] you can feel them and remember.”* [27]

##### Continuing bond

*“So it still up to this day motivates me to keep going, and I say it’s like as if my baby is here laying on me. I put his teddy bear on my chest and lay there and I just I listen.” *[30]

##### Establish connections with family members

*“Here’s one way to describe your brother by showing a picture and sharing it and explaining who he is.”* [26]* Especially to help family members who can’t come “… help your family … I mean, family that wasn’t able to be here … So they can still have a connection to that child.”* [31]

#### Creation of positive memories

Photographs serve as a means to establish a lasting and concrete record, preserving irreplaceable moments and mitigating parents’ apprehension about forgetting. During the photography session, parents were afforded the opportunity to interact with their children. Additionally, some parents crafted shared memories by reading to their children. Such special occasions permitted parents and their children to engage in various familial activities and spend quality time together.

##### Taking photos creates positive memories for parents

*“[Our photographer] had the knack of transporting us into an environment of normalcy, where we were just living hospital, hospital, hospital, but when she came in it was like, oh this is what normal people do, that’s right, you have photographs of your children taken whether it’s at Sears or JC Penney, you know.... There was a gift in that moment of just creating a very normal moment for us.”* [26]

##### Reading to children provided positive memories for parents, as well as ways for parents to care

*What we found that was really good was to feel like you were doing something. To feel like you were interacting and doing something with your baby.* [22]

#### Permission to share

Parents yearn for the opportunity to engage in conversations and share narratives about their deceased children. These tangible and intangible conduits facilitate discussions that enable parents to maintain a connection with their children, effectively keeping their relationship alive. By presenting a photograph or an object and sharing it while providing explanations about the child’s identity, parents can perpetuate their bond with their departed loved one.

##### A way to start a conversation

*“That’s one way to introduce the subject, because it’s always a little like, “When do we tell them we have a dead child?” Because it’s not the easiest thing to bring up, yet it’s really important for us because he’s still so much of our family.”* [26]*.*

##### Talk about kids without pressure

*“We felt like creating the legacy artwork allowed us to talk about tough things without feeling pressured. It helped us put our feelings out there and to express ourselves. Sometimes, you just cannot put your pain into words. So instead of burying them, we used the art to share them with each other.”* [19]

##### Parents want to share things about their children

*“ … I just remember seeing her story [referring to another participant] … and I just remember praying for her and for you guys before I even met them. And it’s just amazing how God puts people in your path in your life.”* [31]

#### Reduces sadness

Legacy art serves as a valuable medium for facilitating the healthy expression of repressed emotions intertwined with grief. Employing legacy as a means to aid parents in their enduring grieving process enables them to articulate a spectrum of emotions encompassing sadness, anger, resentment, joy, and pride. This capacity to seamlessly integrate the memory of their lost child into their everyday existence represents a state of equilibrium for the majority of families. Such integration not only fosters healing but also facilitates their progression beyond the profound loss they have experienced.

##### Photos can relieve the parents’ sadness

*“It was a lot easier than I expected. I did not completely come apart. This conversation made me realize that his legacy lives on as we speak. I just want to say thank you. This has filled my heart up, this talking, you know, reminiscing through the stories. I’m smiling really big right now—Parent.”* [19]

##### Provide the freedom to grieve

*“It’s nice to know they’re always there, if we need to... and sometimes you do want to feel sad, and so sometimes, looking at the images is a good way to let yourself have the permission... to feel sad right now. (Mother)”* [26]

##### To enable parents to return to their children’s memories and affirm their lives

*“I can touch and see his fingerprint and push and hear the heartbeat…so it’s like I see him, my baby, right here.” *[30]

#### Being a parent

Parents emphasize the paramount significance of actively participating in the formulation of a personalized legacy. The act of physically being present with their baby, engaging in tactile contact and cuddles, constitutes a vital and foundational aspect of parenthood. This involvement facilitates the creation of positive memories with their child, simultaneously aiding them in transitioning into their role as parents. For a substantial number of parents, photographs serve as tangible proof of their child’s existence, thereby validating their status as parents.

##### Access to children

*“(the nurse) took him out, and I nursed him. Thank god. And I nursed him for… probably about 3 h.”* [22]

##### Evidence that the child was there

*“It helps me a lot because I don’t want to just forget about her…[to] be able to look up and see all of her pictures on the wall, it puts a smile on, at least knowing that she can always be there.”* [30]

##### Affirmation of parenthood

*“It’s a validation of being a parent... that this person was here on this planet, and that he lived. He was here very briefly, he had a huge impact on our lives, and that presence is recognized and celebrated in the pictures.”* [26]

### Best practices for memory making

#### The timing and place of memory making

The NICU may not be conducive to memory-making practices for parents due to the constraints imposed upon them. Parents contend that the timing of memory-making practices can significantly influence the grieving process. Early interventions offer parents more opportunities to spend time with their children and create tangible and intangible memories. However, some parents harbor concerns that early interventions may impact their hope for their children’s treatment.

##### Not in the NICU

*“I feel the same way. I love the hospital, but I hate coming up here. I’ve had a lot of loss up here.”* [31]

##### The NICU has a limited environment

*“Tom [Baby’s father] and I emm [pause] got to help with Patrick like we never [pause] got to wash him or bathe him [pause] or do anything with him until he died [pause] we didn’t get to hold him or hug him and kiss him.”* [27]

##### Get to hospice early

*“If maybe we had been referred sooner [pause] we would have had a chance to do that [memory making sessions at home with her child participating].”* [27]

##### Memory making measures may be more appropriate after all treatment options have been exhausted

*“You know it’s going to come [the point when no further curative options existed]. And so when the moment came, she pulled me to the side and said, ‘Listen, we’re going to put him to sleep, and I want to see if you suggest to do a fingerprint and a heartbeat,’ she said, ‘so you can always have that.’”* [30]

#### Understand the different types of memory making

Parents express a strong desire to acquaint themselves with diverse memory-making methods, encompassing tangible artifacts like photographs, videos, necklaces, and handprints. Concurrently, during their interactions with their children, parents aspire to forge an intangible legacy through activities such as bathing and cuddling.

##### The child’s hair necklace

*“She was able to cut locks of her son’s hair before he died, which she had made into a necklace.”* [28]

##### Leave handprints

*“The daddy/mommy/me handprint legacy artwork was very meaningful because it was all of us working on that art as a family. We were creating memories as a family. So that helps to fill a small void in my heart, knowing we have those memories as a family with him.”* [19]

##### Have photos of the children

*“I mean, if it wasn’t for my sister, we would not have had the videos and pictures, and I cherish those.”* [31]

##### Digital stories

*“Digital stories could help future families that might undergo similar situations, regardless if baby ultimately died or survived.”* [31]

##### Touch the child

*“And I just got to touch them and study their feet and study their faces. And just soak them in, not forgetting one little detail. She adds: That was really amazing. And that was really important. I can still close my eyes and see their faces 8 years later. So um, yeah, that was really important.”* [22]

#### Respect the needs of parents

Parents desire open and comprehensive communication with healthcare providers, seeking their guidance throughout the memory-making process. Particularly, for parents facing the impending loss of their children, they hope healthcare providers will honor their preferences and facilitate the preservation of their children’s memories in a personalized manner. In addition, some participants described how their religious and spiritual framework influenced their perceptions of heritage and experiences of grief.

##### Taking pictures for the parents

*“This was the only way, we don’t have any other option.... It was either this, or rely on our memory, and I think we both felt that we wanted something more permanent than just our memory of him.”* [26]

##### Touch and hug the child

*“I wanted to see him. I wanted to see his toes and his fingers, because I hadn’t really… as much as I had touched him, I hadn’t really held him.”* [22]

##### Spirituality

*“there’s no time in heaven. So, when they get there, they meet Jesus, and you are already there. We’re already there. I’m already with her. She’s not alone. And that gives me great peace to know she is not alone.”* [28]

##### Healthcare support for parents

*“…couldn’t have gotten through those forty-five days without the nurse staff…They just, they just, would explain as much as you wanted to hear. They would just, stand there with you…they were, you know, taking care of the baby but also taking care of you mentally…we still talk to them on Facebook and stuff like that.”* [28]

### Obstacles to memory making

#### Stress when initially receiving the memory making intervention

Parents are deeply immersed in the news of their child’s death, and in such circumstances, they perceive an immediate engagement with memory-making activities as emotionally distressing, placing significant stress upon them.

##### Hard to choose

*“In that state of shock and drowning grief making decisions is really hard, and so it is easier to say no to everything than to have to think about it…[parents] don’t know what to do.”* [32]

##### Parents are stressed when babies are unstable

*“They really really encouraged us to get him out and hold him. And that I value a lot. Because at the time I didn’t want to, because it stressed him out so much. But looking back I wish we had have had more experiences holding him and cuddling him. So that was really good.”* [22]

##### Parents’ physical condition affects the contact with children

*“It’s that feeling of helplessness and hopelessness when you’re standing there. You know, every time you touch him, his blood pressure would either drop through his boots, or go through the roof.”* [22]

#### Misunderstand

Many parents exhibit ambivalence when confronted with various forms of memory making, primarily due to a lack of understanding. Furthermore, the diversity of memory-making options available for different families can be a source of frustration and confusion for parents.

##### Resistance to posthumous photography

*“I thought they were out of their mind when the question was raised….In the end I was grateful.”* [32]

##### Antiphotography

“In the beginning my entire family was against us having a photographer present” [22]

##### Bewilderment

*“That can be a little uncomfortable, being like, ‘oh my child life specialist says,’ and you don’t want to ever feel like, ‘why did [that other family] get this’ or…[not know] what the criteria is.”* [30]

#### Underpreparation

Parents perceive separation as an emotionally distressing experience, even when it is deemed medically necessary. This separation exerts a profound influence on parental interactions with their infants, limiting the time parents can spend together with their child. Consequently, parents often find themselves ill-prepared for the imminent loss of their child.

##### Helplessness

*“It’s that feeling of helplessness and hopelessness when you’re standing there. You know, every time you touch him, his blood pressure would either drop through his boots, or go through the roof.”* [22]

##### Insufficient time

*“We felt robbed of our time with our son…the hospital staff made it seem very limited….Many more pictures could have been taken if we had been afforded an opportunity.”* [32]

##### Inadequate mental preparation

*“I didn’t think about taking videos. I just wasn’t in my … neither one of us … I wish I would have….”* [31]

## Discussion

This study conducted a systematic review and synthesis of nine qualitative studies with the aim of delving into the experiences and perspectives of memory making among bereaved parents. Concerning memory making, parents strongly endorse its value and significance. They articulate their individual needs and perspectives on memory making, shedding light on the challenges they encounter while engaging in memory-making practices. In this study, parents of infants and parents of children who died of cancer were included to gain a more comprehensive understanding of bereaved parents’ perceptions of memory making. It is noteworthy that the bulk of insights obtained from the studies included primarily derive from mothers’ perspectives. Given the reliance on maternal accounts, a noticeable gap exists within the research literature that warrants attention. Future research endeavors should prioritize exploring fathers’ perspectives and viewpoints regarding memory creation. This approach is crucial to comprehensively understand the collective impact of memory making, encompassing both parental viewpoints.

This study underscores the strong acceptance of memory making among bereaved parents. The majority of parents in our study expressed a positive inclination towards memory making as a means to establish a profound connection with their deceased children. These objectives hold significant sentimental value for them, serving as a conduit for creating lasting memories and nurturing emotional bonds with their departed loved ones. This connection not only encompasses parents’ interactions with their children but also extends to the involvement of other family members in cherishing the memories of these children. Notably, Meert’s research revealed that parents regarded maintaining a connection with their children as their primary spiritual need [33], a source of solace in times of adversity, and a means to find comfort. The loss of this connection can potentially lead to complex grief and other mental health challenges for parents [34].

Sim’s investigation [35] further emphasizes that parents’ inability to be with their children at the end of life becomes a source of profound regret. In our study, various memory-making methods such as photographs, reading, narrative stories, and videos played a pivotal role in helping parents and children create positive memories. These activities allow parents to reaffirm their own identities and constitute an integral aspect of their parenthood experience. This affirmation is particularly salient in the context of NICU, where parents find solace in caring for their infants. Abraham’s research [36] also highlights parents’ strong desire to fulfill their parental roles and provide warmth and support to their dying infants. Parents benefit significantly from the opportunity to engage in activities like seeing, holding, washing, and dressing their babies, which validates their role as parents [37]. These parents had significantly limited access to their babies in comparison to parents whose children passed away due to cancer. They appeared to exhibit a heightened inclination to nurture their babies and create more enduring memories, particularly through tangible objects.

Parents can preserve valuable memories by retaining items such as photographs, necklaces, handprints, and more related to their children. These tangible objects are viewed as extensions of the deceased child and serve as tangible evidence for parents that their child once existed. This perspective aligns with Thornton’s research, which suggests that items connected to the child facilitate the expression of grief by parents [38], a finding consistent with our own research. In adult intensive care units (ICU) [39], participants reported that these items triggered vivid recollections of their deceased loved ones. While the initial exposure to these items may evoke grief, parents often come to appreciate their significance over time. According to the dual process model [40], engaging with these items provides an outlet for parents to express their grief, potentially reducing the risk of post-traumatic stress disorder and complex grief.

While our research indicates that parents highly value memory making, it is important to note that no clinical studies have definitively established a causal relationship between items such as photos and necklaces and the reduction of parental trauma. Furthermore, during the COVID-19 pandemic, access to bereavement care services significantly dwindled, with parents’ experiences clearly influenced by resource allocation [41]. This has sparked a debate on how to involve families in maintaining contact with their deceased children.

This study has identified varying perspectives among family members regarding the commencement and location of memory-making practices. Riegel’s research [42] emphasizes that the applicability of memory making as a bereavement intervention is not constrained by the cause or place of death. Therefore, the timing and setting of memory-making activities should be determined by the bereaved parents, with healthcare providers playing a crucial role in offering guidance. Establishing rapport and fostering open communication with bereaved parents early in the process can assist clinicians in gauging the optimal moment to introduce memory-making practices [30]. Beiermann’s work underscores the significance of educating caregivers on successful intervention implementation. This education encompasses determining the appropriate timing to approach the family, gauging family acceptance of withdrawal from treatment to initiate discussions, and articulating the offering of the memory legacy [43]. At the end of their child’s life, parents often find themselves in profound grief, unaware of the long-term significance of bonding and memory in the bereavement process, and uninformed about available memory-making options. Our research has revealed that many family members are uncertain about the mechanics of memory creation and require guidance in selecting suitable approaches. Parents express a desire for memory-making options to be provided by healthcare providers [32], with a need for compassionate and impartial support that refrains from imposing personal biases. Therefore, healthcare providers should ensure the availability of diverse memory-making choices, including the provision of professional photographers and memory boxes [44].

When initiating memory-making practices for parents, it is imperative to acknowledge and respect the diverse needs, cultural backgrounds, and spiritual beliefs inherent in various families. Within the study, certain parents refrained from capturing photographs of their deceased children due to cultural considerations, deeming it culturally inappropriate. They regard contact with deceased children as taboo, associating it with potential impacts on future fertility, leading to stigmatization. Other research highlights instances where parents diverging from traditional cultural norms by engaging in rituals involving deceased children encountered conflicts [45]. In specific regions, stillbirths are often construed as indicative of misconduct, subjecting parents to public stigma [46]. The prevailing cultural beliefs and practices in these contexts can exacerbate the already challenging experiences of bereaved parents. It’s worth noting that interventions tailored to the needs of bereaved parents in these cultural settings remain an underexplored area of research.

The study reveals that families often encounter barriers when considering the adoption of memory-making practices. Some families may decline or misunderstand these practices due to delayed access to memory-related items or a lack of awareness about the availability of healthcare professionals. This observation aligns with prior research findings [47]. Further investigation into the preferences and reasons for refusal of memory production among families is essential to inform educational guidance. The implementation of a standardized communication strategy, such as the SPIKES model (Setup, Perception, Invitation, Knowledge, Emotions, Strategy), can enhance education and facilitate reflection and practice through simulation [48].

In our study, bereaved parents displayed a generally positive attitude toward memory making. However, it is noteworthy that during the initial exposure to memory-making practices, parents may exhibit some ambivalence. This ambivalence may arise due to uncertainty regarding the prognosis of their child, emotional distress, and a lack of available support personnel [31]. Insufficient parental preparation can significantly impact the development of memory making, with parents who missed out on opportunities expressing deep regret and a sense of loss [49]. Rigel’s study [50] pointed out that nurses, in comparison to doctors, often perceive themselves as the healthcare providers who spend the most time with patients and are better equipped to meet the needs of family members. Nurses can enhance parental readiness for the loss of a child by providing personalized guidance and support. Nevertheless, some parents reported not receiving counseling regarding the available personal care options for their infants [39]. Parental readiness for a child’s death can be improved through the provision of information to parents and by accessing support from healthcare providers or peers [16].

## Implications for practice

Our research underscores the necessity for parents to receive proactive guidance and practical assistance when engaging in memory-making activities with their children. Healthcare providers are ideally positioned to facilitate and support bereaved parents in this endeavor. This study highlights the crucial role of empathetic healthcare providers in providing the needed support to bereaved parents. Healthcare providers can actively encourage parents to engage in physical contact with their children, encompassing activities such as cuddling, touching, and bathing. Parents should have access to high-quality and compassionate bereavement support, ideally delivered by trained professionals. Psychosocial support should be administered to families with a high degree of compassion and sensitivity. To best meet the needs of bereaved families, a flexible and personalized approach to bereavement care should be developed and implemented by dedicated professionals committed to patient-centered care.

## Conclusion

The study aims to investigate the experiences and perspectives of parents engaged in memory making. Our research findings indicate that bereaved parents not only acknowledge the importance of memory making but also perceive it as increasingly meaningful over time, despite initial stress. However, during the memory-making process, some parents miss opportunities due to a lack of understanding, inadequate preparation, and limited available assistance, leading to subsequent regret. For parents, memory making serves as an effective means to connect with their children and navigate the grieving process. To offer effective bereavement support to parents, a multifaceted approach should be adopted, encompassing the provision of diverse options and bereavement counseling. Furthermore, there is a pressing need for an in-depth exploration of memory-making practices to gain a more nuanced understanding of their impact on parents’ memory experiences. Equally important is the necessity to provide healthcare providers with training in memory making and offer timely guidance to bereaved parents.

## Strengths and limitations

Our study synthesized the experiences and perspectives of bereaved parents concerning memory making, delved into their potential needs, and elucidated the influencing factors inherent in the memory-making process. These findings offer valuable insights for the development of future effective intervention strategies. However, certain limitations of this study should be acknowledged. Firstly, the study exclusively drew from English literature, omitting potential insights from grey literature, which could introduce a degree of bias. Furthermore, given the cultural and policy variations across different regions, researchers may hold divergent perspectives and interests. Additionally, the needs of bereaved parents can be profoundly shaped by regional cultural differences, potentially resulting in disparate outcomes. Most of the insights in our study are derived from mothers, and it is not yet known whether forms of memory making are equally effective for fathers. Future research needs more research on fathers in order to incorporate their support needs.

### Supplementary Information

**Additional file 1.**

## Data Availability

All data generated or analysed during this study are included in this published article [and its supplementary information files].
