# Supplementary material for: Bereaved parents’ perceptions of memory making: a qualitative meta-synthesis
Source: BMC Palliat Care. 2024 Jan 25;23:24. doi: 10.1186/s12904-024-01339-0 (PMC10809512; doi:10.1186/s12904-024-01339-0)
Supplement: Supplementary file 1 — Additional file 1. [file 12904_2024_1339_MOESM1_ESM.docx]

**Bereaved parents' perceptions of memory making: A qualitative meta-synthesis**

**Supplementary appendix**

**Pubmed: 357 results**

#1 Hospice Care [mh] OR Care, Hospice [tiab] OR Hospice Program* [tiab] OR Program, Hospice [tiab] OR Bereavement Care [tiab] OR Care, Bereavement [tiab]

#2 Terminal Care [mh] OR Care, Terminal [tiab] OR End of Life Care [tiab] OR End-Of-Life Care [tiab] OR Care, End-Of-Life [tiab] OR End-Of-Life Cares [tiab]

#3 Parents [mh] OR Parent [tiab] OR Parent [tiab] OR Parenthood Status [tiab] OR Status, Parenthood [tiab] OR Step-Parent* [tiab] OR Stepparent* [tiab] OR Parental Age* [tiab] OR Age, Parental

#4 intervention* OR strateg*

#5 #1 OR #2

#6 #5 AND #3 AND #4

**EMBASE: 580 results**

#1 'hospice care'/exp OR 'hospice care' OR 'care, hospice':ti,ab,kw OR 'program, hospice':ti,ab,kw OR 'care, bereavement':ti,ab,kw

#2 'terminal care'/exp OR 'terminal care' OR 'care, terminal':ti,ab,kw OR 'end of life care':ti,ab,kw OR 'care, end-of-life':ti,ab,kw

#3 #1 OR #2

#4 'parents'/exp OR parents OR parent*:ti,ab,kw OR stepparent*:ti,ab,kw

#5 intervention*:ti,ab,kw OR strateg*:ti,ab,kw

#6 #5 AND #3 AND #4

**Cochrane Library: 565 results**

#1 MeSH descriptor: [Hospice Care] explode all trees

#2 (Care, Hospice):ti,ab,kw OR (Hospice Program*):ti,ab,kw OR (Bereavement Care):ti,ab,kw

#3 MeSH descriptor: [Terminal Care] explode all trees

#4 (Care, Terminal):ti,ab,kw OR (End of Life Care):ti,ab,kw

#5 #1 or #2 or #3 or #4

#6 (Parent*):ti,ab,kw

#7 (intervention*):ti,ab,kw OR (strateg*):ti,ab,kw

#8 #5 and #6 and #7

**Web of Science :968 results**

TS=(((Hospice Care OR Hospice OR Hospice Program* OR Bereavement Care OR Terminal Care OR End of Life Care OR End-Of-Life Care OR End-Of-Life Cares) AND (Parents OR Parent OR Parent OR Step-Parent* OR father OR mother ) AND (intervention* OR strateg*))

**CINAHL：13 results**

#1 (MM "Hospice Care")  or SU （Care, Hospice ） or (MM "Terminal Care+")  or SU （Care, Terminal ）

#2 (MM "Parents+") OR SU Parent*

#3 SU intervention* OR SU strateg*

#4 #1 AND #2 AND #3

**Wiley: 352 results**

#1 (“Hospice Care” OR “Hospice Program*” OR “Bereavement Care” OR “Terminal Care” OR “End of Life Care” OR “End-Of-Life Care*”)
#2 (“Parent*” OR “Stepparent*” OR “father” OR “mother”)

#3 (“intervention*” OR “strateg*”)

#4 #1 AND #2 AND #3

**PsycINFO：5 results**

#1 (MM "Hospice Care")  or SU （Care, Hospice ）or (MM "Terminal Care+")  or SU （Care, Terminal ）

#2 (MM "Parents+") OR SU Parent*

#3 SU intervention* OR SU strateg*

#4 #1 AND #2 AND #3
